# Enhancing Digital Health Interventions for Medication Adherence: Considerations for Broader Applicability and Long-Term Impact

**DOI:** 10.2196/69204

**Published:** 2025-03-14

**Authors:** ShanShan Du, Yining Zhao

**Affiliations:** 1 Renhe Rehabilitation Hospital Hubei China; 2 The First Hospital of Shanxi Medical University Shanxi China

**Keywords:** mobile apps, digital health, atrial fibrillation, anticoagulants, medication adherence, mobile phone

We appreciate the opportunity to read the recently published article by Yoon et al [1], titled Smartphone App for Improving Self-Awareness of Adherence to Edoxaban Treatment in Patients With Atrial Fibrillation (ADHERE-App Trial): Randomized Controlled Trial. The study addresses an important clinical challenge—improving medication adherence in atrial fibrillation patients using digital health interventions. While the findings are valuable, we would like to highlight some additional limitations and considerations that we believe warrant further discussion.

First, the study population was limited to individuals familiar with Android smartphones and the Korean language, likely excluding older adults with lower digital literacy. This presents a significant limitation, as older adults, who often make up a substantial proportion of patients requiring anticoagulation therapy, frequently struggle to adopt and effectively use smartphone-based interventions. For this demographic, navigating apps, managing settings, and understanding instructions on a smartphone can already be challenging, and unfamiliarity with technology exacerbates these difficulties. Such barriers may significantly restrict the practicality and accessibility of these interventions for those who stand to benefit the most. Expanding the target population to include individuals with varying levels of digital literacy and providing tailored support for older adults would greatly enhance the inclusivity and real-world applicability of future research. Additionally, the exclusive focus on edoxaban limits the generalizability of the findings to patients using other anticoagulants with differing dosing regimens and side effects. Broadening the intervention to encompass a more diverse patient population and various anticoagulants would strengthen the external validity and clinical relevance of the study.

Second, the 6-month follow-up period is another critical consideration, as adherence is a dynamic behavior that often fluctuates over time. While the study demonstrated short-term benefits, long-term follow-up is essential to determine whether these improvements are sustained and lead to meaningful clinical outcomes, such as reduced stroke or bleeding risks [2]. Moreover, relying on pill counts as the primary adherence metric, while practical, does not account for the accuracy of timing or dosage. Using more precise tools, such as electronic medication event monitoring systems, could provide deeper insights into adherence patterns and their clinical implications.

Lastly, although the intervention design is innovative, it could be improved to enhance engagement. Incorporating personalized feedback or adaptive features tailored to individual adherence behaviors might increase user retention and app effectiveness, especially given the higher dropout rates observed in the intervention group. Additionally, integrating the app with complementary strategies, such as caregiver notifications or telemedicine support, could make the intervention more accessible to patients with varying levels of technological proficiency. Addressing these limitations would allow future research to more comprehensively evaluate the app’s practicality and clinical impact.

In conclusion, while Yoon et al [1] provide a well-executed study demonstrating the potential of smartphone apps to improve medication adherence, addressing the above considerations could further enhance the clinical relevance and applicability of their findings. We commend the authors for their contributions to this evolving field and look forward to future research addressing these important aspects.
